# Supplementary figures and images for: The effects of mental fatigue on explicit and implicit contributions to visuomotor adaptation
Source: PLoS One. 2024 Aug 15;19(8):e0307739. doi: 10.1371/journal.pone.0307739 (PMC11326645; doi:10.1371/journal.pone.0307739)

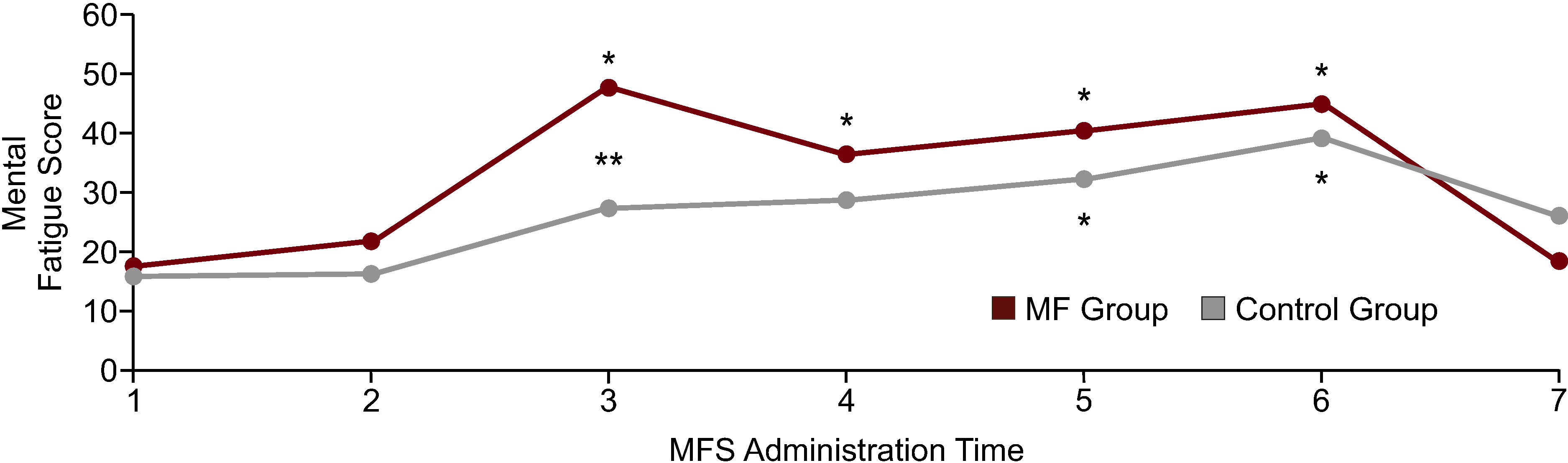

Supplement: S1 Fig — Mental fatigue scores for the MF group (burgundy) and Control group (grey) reported across the experiment. Asterisks (*) represent significant differences relative to initial levels of mental fatigue reported at Time 1 (p < 0.05). Double asterisks (**) represent significant differences between groups (p < 0.05). (TIF) [file pone.0307739.s001.tif]

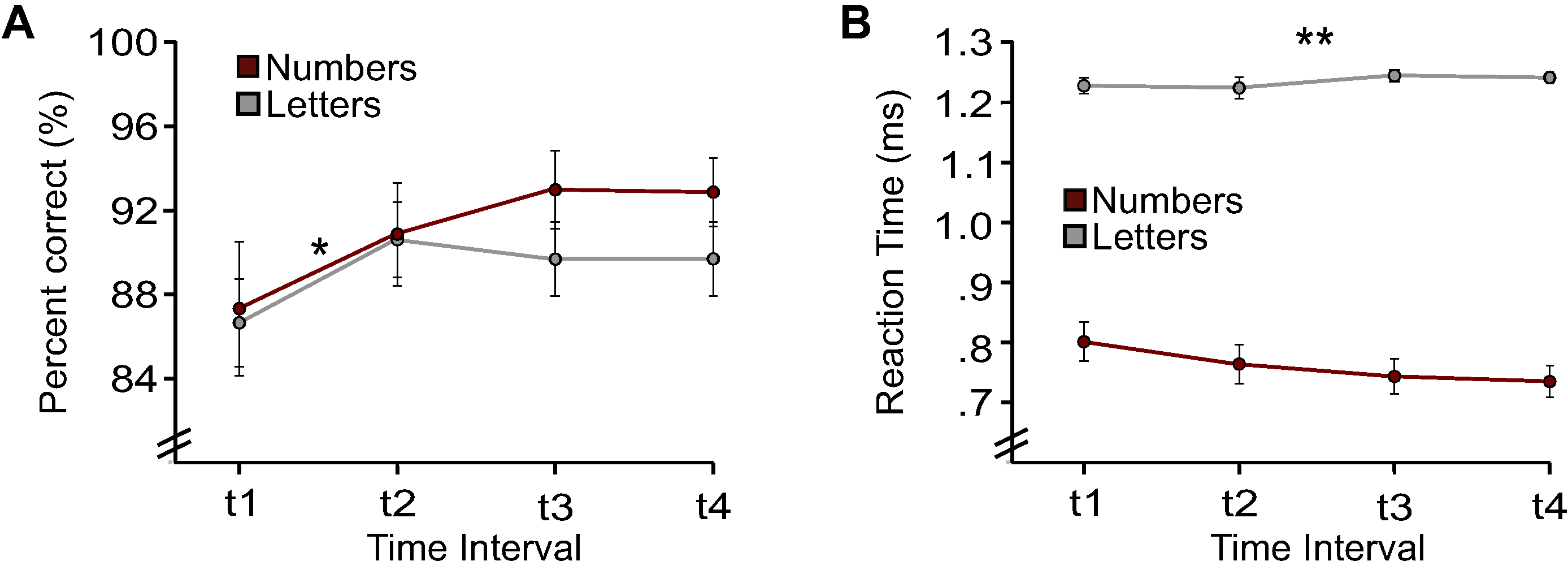

Supplement: S2 Fig — Mean performance across the 4 time intervals of the TLDB task for the Mental Fatigue group, separated by numbers (garnet) and letters (grey). (A) Percentage of correct responses and (B) mean reaction time in seconds. Error bars represent standard error of the mean. Asterisks (*) represent significant differences between consecutive time intervals in (A) (p < 0.05). Double asterisks (**) represent significant differences between stimuli in (B) (p < 0.05). (TIF) [file pone.0307739.s002.tif]
