# Supplementary material for: The effects of mental fatigue on explicit and implicit contributions to visuomotor adaptation
Source: PLoS One. 2024 Aug 15;19(8):e0307739. doi: 10.1371/journal.pone.0307739 (PMC11326645; doi:10.1371/journal.pone.0307739)
Supplement: S1 File — (DOCX) [file pone.0307739.s003.docx]

**Supplementary File:** **The effects of mental fatigue on explicit and implicit contributions to visuomotor adaptation**

David Apreutesei^1^ & Erin K. Cressman^1^

^1^ School of Human Kinetics, Faculty of Health Sciences, University of Ottawa, Ottawa, Ontario, Canada

## **Data Analyses and Results**

### **Mental Fatigue Scale (MFS)**

Responses on the MFS were determined for each participant for each of the 7 times it was completed. Responses for each participant were determined to be the value in millimeters between 0 to 100 according to the location on the continuous line at which participants indicated their current level of mental fatigue, where 0 represented no mental fatigue and 100 represented extreme mental fatigue. A 2 Group (MF vs. Control) x 7 Time (i.e., each time the MFS was completed) mixed analysis of variance (ANOVA) with repeated measures (RM) on the last factor was conducted to establish changes in mental fatigue over the course of the experiment within and between groups.

Reports of mental fatigue over the course of the experiment are displayed in Fig S1. A similar figure is presented in the original manuscript. In the Supplementary File we report changes in mental fatigue over the course of completing the rotated reach training trials for both groups. ANOVA revealed that mental fatigue did differ across Time (F(3.3, 124.9) = 29.470, *p < 0.001*), and there was a significant Group x Time interaction (F(3.3, 124.9) = 5.111, *p = 0.002*). Mental fatigue did not differ from initial values for the Control group until the end of the second rotated training block (Time 5: X̅ = 32.6, SE = 4.8), at which time it increased significantly from Time 1 (X̅ = 16.5, SE = 4.1; *p = 0.006*). Mental fatigue continued to be increased following the third rotated training block relative to Time 1 (Time 6: X̅ = 39.3, SE = 5.6; *p < 0.001*). Mental fatigue then decreased following the rest period do that it did not differ from initial levels (Time 7: X̅ = 26.5, SE = 4.7; *p = 0.381*).

As for the MF group, mental fatigue remained evaluated relative to Time 1 (X̅ = 18.2, SE = 3.3) following each of the three blocks of rotated reach training trials (Time 4: X̅ = 36.7, SE = 4.1; Time 5: X̅ = 40.6, SE = 4.7; Time 6: X̅ = 45.0, SE = 5.4; all *p < 0.001*). There was a small decrease in mental fatigue reported following completion of the first rotated training block (Time 4) relative to completing the TLDB task (Time 3: X̅ = 47.7, SE = 5.8; *p = 0.040*), showing a decay of mental fatigue induced by the TLDB within minutes (see Jacquet et al., 2011 for similar results). Mental fatigue then decreased again for the MF group following the rest period, at which time mental fatigue did not statistically differ from initial mental fatigue levels reported by the MF group (Time 7: X̅ = 19.0, SE = 3.0; *p = 1*). Reports of mental fatigue only differed between groups at Time 3 (*p = 0.013*), following completion of the TLDB task or documentary watching.

**Fig S1. Mental fatigue reports across the experiment.** Mental fatigue scores for the MF group (burgundy) and Control group (grey) reported across the experiment. Asterisks (*) represent significant differences relative to initial levels of mental fatigue reported at Time 1 (*p < 0.05*). Double asterisks (**) represent significant differences between groups (*p < 0.05*).

## **Time Load Dual Back Task (TLDB)**

### **Response Accuracy and Reaction Time (RT)**

Performance on the TLDB task for the MF group was evaluated by assessing response accuracy and time of responses (i.e., reaction time) relative to stimulus presentation over the duration of the TLDB task. This analysis was performed for both types of stimuli presented (i.e., letters and digits). Percent accuracy was the percentage of correct responses based on stimulus type (i.e., pressing (or not pressing) the spacebar when the current letter had appeared (or not appeared) previously; pressing the “2” (or “3”) key when an even (or odd) number was shown). Reaction time for all responses was measured as the time taken to press a key after the presentation of each stimulus. Percent accuracy and mean reaction time for the letter and digit responses were determined for 4 time intervals (t1, t2, t3 and t4), with each time interval representing 25% of the total number of trials completed (i.e., 315 trials of the total 1260 trials), and analyzed within a 2 Stimulus Type (letters vs. digits) x 4 Time Interval (t1, t2, t3, vs. t4) RM ANOVA. Percentage data were arcsine transformed before undergoing analyses.

Results related to the TLDB task are shown in Fig S2. ANOVA revealed a significant main effect of Time Interval with respect to percent accuracy (F(2.1, 40.8) = 9.107, *p < 0.001*), and a significant Stimulus Type by Time Interval interaction (F(3, 57) = 3.695, *p = 0.017*). Percent accuracy did not change for letters over the course of the TLDB task (Time Interval 1: X̅ accuracy = 86.6%, SE = 2.1%; Time Interval 4: X̅ accuracy = 89.6%, SE = 1.8%; *p = 0.293*). Percent accuracy did change for numbers, such that accuracy improved from the first to the second time interval (Time Interval 1: X̅ accuracy = 87.3%, SE = 3.2%; Time Interval 2: X̅ accuracy = 90.8%, SE = 2.4%; *p = 0.008*). There was no further improvement in accuracy across the third and fourth time intervals (both *p > 0.105*). Accuracy for numbers was greater than letters in the third (*p = 0.008*) and fourth time intervals (*p = 0.041*).

As seen in Figure S2 B, reaction time was higher overall when responding to letters compared to numbers (F(1, 19) = 183.540, *p < 0.001*). ANOVA also revealed a significant Stimulus Type x Time Interval interaction (F(1.9, 36.3) = 5.180, *p = 0.011*). However post hoc analysis indicated that reaction time for letters and numbers remained constant, with no significant changes between the first and fourth time intervals for letters (Time Interval 1: X̅ RT = 1.22 sec, SE = 0.01; Time Interval 4: X̅ RT = 1.24 sec, SE = 0.01; *p = 1*) and numbers (Time Interval 1: X̅ RT = 0.80 sec, SE = 0.03; Time Interval 4: X̅ RT = 0.73 sec, SE = 0.03; *p = 0.075*).

##### **S2 Fig. Percent accuracy and mean reaction time across the TLDB task.** Mean performance across the 4 time intervals of the TLDB task for the Mental Fatigue group, separated by numbers (garnet) and letters (grey). **(A)** Percentage of correct responses and **(B)** Mean reaction time in seconds. Error bars represent standard error of the mean. Asterisks (*) represent significant differences between consecutive time intervals in **(A)** (*p < 0.05*). Double asterisks (**) represent significant differences between stimuli in **(B)** (*p < 0.05*).
